# Mysteriously rapid rise in Legionnaires’ disease incidence correlates with declining atmospheric sulfur dioxide

**DOI:** 10.1093/pnasnexus/pgae085

**Published:** 2024-03-12

**Authors:** Fangqun Yu, Arshad A Nair, Ursula Lauper, Gan Luo, Jason Herb, Matthew Morse, Braden Savage, Martin Zartarian, Meng Wang, Shao Lin

**Affiliations:** Atmospheric Sciences Research Center, University at Albany, State University of New York, Albany, NY 12226, USA; Atmospheric Sciences Research Center, University at Albany, State University of New York, Albany, NY 12226, USA; New York State Department of Health, Bureau of Water Supply Protection, Albany, NY 12223, USA; Atmospheric Sciences Research Center, University at Albany, State University of New York, Albany, NY 12226, USA; Atmospheric Sciences Research Center, University at Albany, State University of New York, Albany, NY 12226, USA; New York State Department of Health, Bureau of Water Supply Protection, Albany, NY 12223, USA; New York State Department of Health, Bureau of Water Supply Protection, Albany, NY 12223, USA; New York State Department of Health, Bureau of Water Supply Protection, Albany, NY 12223, USA; School of Public Health and Health Professions, University at Buffalo, State University of New York, Buffalo, NY 14214, USA; School of Public Health, University at Albany, State University of New York, Albany, NY 12144, USA

**Keywords:** aerosols, legionellosis, SO_2_, biological transport, cooling towers

## Abstract

Legionnaires’ disease (LD) is a severe form of pneumonia (∼10–25% fatality rate) caused by inhalation of aerosols containing *Legionella*, a pathogenic gram-negative bacteria. These bacteria can grow, spread, and aerosolize through building water systems. A recent dramatic increase in LD incidence has been observed globally, with a 9-fold increase in the United States from 2000 to 2018, and with disproportionately higher burden for socioeconomically vulnerable subgroups. Despite the focus of decades of research since the infamous 1976 outbreak, substantial knowledge gaps remain with regard to source of exposure and the reason(s) for the dramatic increase in LD incidence. Here, we rule out factors indicated in literature to contribute to its long-term increases and identify a hitherto unexplored explanatory factor. We also provide an epidemiological demonstration that the occurrence of LD is linked with exposure to cooling towers (CTs). Our results suggest that declining sulfur dioxide air pollution, which has many well-established health benefits, results in reduced acidity of aerosols emitted from CTs, which may prolong the survival duration of *Legionella* in contaminated CT droplets and contribute to the increase in LD incidence. Mechanistically associating decreasing aerosol acidity with this respiratory disease has implications for better understanding its transmission, predicting future risks, and informed design of preventive and interventional strategies that consider the complex impacts of continued sulfur dioxide changes.

**Significance Statement**

Legionnaires’ disease (LD) is a severe lung infection caused by the *Legionella* bacteria. It is a public health concern since its incidence has risen dramatically and inexplicably in recent years. In our study, we find the unexplored factor of declining atmospheric sulfur dioxide concentrations ([SO_2_]) to be associated with increasing LD incidence. We also provide further evidence that cooling towers (CTs) are likely a major source of LD. We find that declining [SO_2_] results in reduced acidity of CT droplets, which can prolong *Legionella* lifetime in infected droplets and thus explain the observed increase in LD incidence. Mechanistically associating decreasing aerosol acidity with this respiratory disease has potential implications for forecasting LD risks and needed interventions considering continued [SO_2_] changes.

## Introduction

Legionnaires’ disease (LD) is a severe form of pneumonia with a hospitalization rate of ∼95% and a fatality rate of ∼10–25% that is caused by multiple bacterial species of the *Legionella* genus (1–3). *Legionella* are found naturally in fresh water such as lakes and streams. The bacteria can become a health concern when they grow and spread in built water systems like cooling towers (CTs), hot tubs, decorative fountains, hot water tanks, showerheads, and sink faucets (4–6). Infection occurs when aerosolized *Legionella* are inhaled, or contaminated water is aspirated and bacteria enter the lungs (7). Owing to the superior reporting standards of the US Centers for Disease Control and Prevention (CDC) and its designation of LD as a nationally notifiable disease, LD cases since 1992 have been well documented in the United States, where we focus this study. Here, the species *Legionella pneumophila* (*Lp*) accounts for ∼90% of cases (8). LD incidence is higher in the summertime, possibly because of increased use of CTs for air conditioning systems and differences in water chemistry and bacterium growth when outdoor temperatures are higher. Those most vulnerable to LD are male, over 50 years of age, have a history of smoking, have chronic respiratory diseases, diabetes, are immunocompromised, and/or minorities (4, 9).

The United States has experienced a dramatic increase in LD incidence over the last two decades, with ∼1,100 cases reported in 2000 and nearly 10,000 cases in 2018 (3, 4). The reason for this increase is unknown but is likely multifactorial (10). Although increased testing may have contributed to the initial rise in reported LD cases in the United States, it does not explain the continued increase in incidence (4). The LD incidence rate in Europe and Ontario, Canada, have been increasing as well in the last two decades (4), both by a factor of ∼5–7. Despite active surveillance and reporting requirements in the United States, cases are thought to be underreported by as much as 77–179% (11, 12). This may be at least partially due, in addition to other reasons such as misdiagnosis as other respiratory illnesses or asymptomatic/mild presentation, to limited access to quality healthcare among vulnerable residents of underserved communities. Additionally, ethnic and racial minorities may be disproportionately impacted, with widening disparities over time (4, 9).

While progress has been made in understanding *Legionella* transmission in the past 45 years since the infamous 1976 outbreak during the Philadelphia convention of the American Legion, substantial knowledge gaps remain (4, 7, 13). First, the vast majority (>90%) of community-acquired cases are sporadic with no known point source of exposure (14). Second, the reason(s) for the rapid increase of LD cases in the United States and other countries over the last two decades remains unknown (2, 4, 15). Population aging and changes in the case definition or available diagnostic tests are unlikely to explain the extent of this increase (4). Possible environmental factors influencing LD cases include relative humidity (RH), temperature (*T*), precipitation, and UV radiation (7, 16–20), but the mechanisms and the magnitude of these influences remain unclear. While these factors may contribute to daily, seasonal, or even interannual variations of LD cases, here we show that they are unable to explain the observed long-term increasing trend. We further demonstrate that this long-term increase in LD is associated with the long-term decline in atmospheric sulfur dioxide concentrations, under which the survival of *Legionella* is potentially prolonged. These findings have implications for assessing the risk of LD in the future (as SO_2_ continues to decline) and designing strategies to counter the LD increase.

## Results

### Trend of LD in the United States, Northeast United States, and New York State

**Fig. 1.**
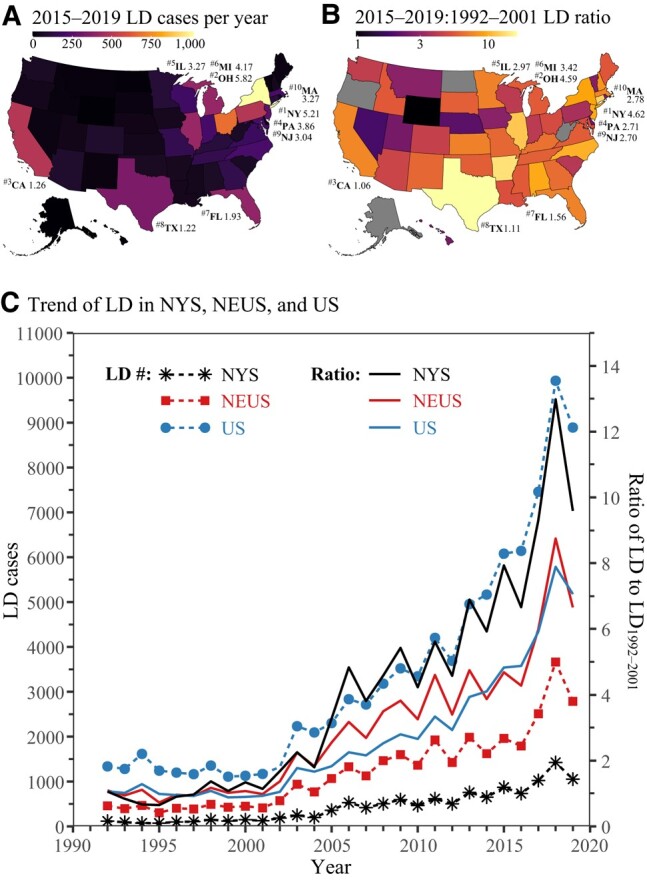
Spatial and temporal variations in LD. A) Statewise LD cases per year during 2015–2019 indicated with color scale (linear). LD incidence ratio (IR, cases per year/100,000 individuals) noted for the top-10 LD case states. B) Statewise LD ratio of cases between 2015–2019 and 1992–2001 indicated with color scale (log_10_). Corresponding increase in LD incidence (i.e. $\Delta \text{IR}={\text{IR}}_{2015-2019}-{\text{IR}}_{1992-2001}$) noted for the top-10 LD case states. Ratios not shown for <2 cases per year in the baseline period of 1992–2001. C) Long-term trends of LD cases and LD ratio (baseline: 1992–2001) from 1992 to 2019 in the United States (US), NEUS, and NYS. CDC data for 2019 may be underestimated because the completeness of the data reported by 23 States (including NYS) is uncertain due to the COVID-19 pandemic (21).

We analyzed reported LD case data from CDC that covers 1992–2019 for all 50 states of the United States. Figure 1 shows the spatial distribution (in all states) and trends of LD cases in the United States, with LD incidence ratio (IR, cases per year/100,000 individuals) and the increase in IR from 1992–2001 to 2015–2019 (i.e. $\Delta \text{IR}={\text{IR}}_{2015-2019}-{\text{IR}}_{1992-2001}$) indicated for top-10 LD case states (i.e. #1–#10) in Fig. 1A and Fig. 1B, respectively. The highest LD burdens are in the Northeast United States (NEUS) and particularly New York State (NYS). Both the top-2 LD cases states (#1 NYS and #2 Ohio) are in NEUS and have IR (NYS 5.21 and Ohio 5.82) and *Δ*IR (NYS 4.62 and Ohio 4.59) much larger than the remaining top-10 cases states. NEUS accounts for one-third (37.2±6.1%) and NYS one-eighth (12.2±3.4%) of LD cases during the entire period. The largest number of cases have been in the recent years (2015–2019) as shown in Fig. 1C. The decade from 1992 to 2001 shows a relatively constant 1259±151 LD cases per year in the United States and is considered as a baseline. Compared to this baseline, the ratio of LD cases during 2015–2019 is shown in Fig. 1B. In addition to NEUS and NYS being high LD burden regions (Fig. 1A), these are also the regions with some of the highest increases in LD burden (Fig. 1B). Figure 1C illustrates the temporal trend for LD with the baseline period followed by rapid rise peaking at 9,933 LD cases in 2018. The trends for both absolute LD cases and IR are consistent for United States, NEUS, and NYS. The LD IR (cases per 100,000 individuals per year) for 2015–2019, which removes the effect of population change, is 5.10× (United States), 5.64× (NEUS), and 8.86× (NYS) relative to the baseline period. Given that the largest increase in LD burden is in NYS, we focus our attention here. We leverage NYS’s high-quality data (including the registered CT database) that was made accessible to us. While the analysis below focuses on NYS only, the findings are expected to be valid in other states and countries.

### The possible factors causing the long-term LD increase

Most transmission of *Legionella* involves aerosolization of bacteria from water into air. Past outbreaks have been linked to sources such as CTs, wastewater treatment plants, public fountains, spas, ice machines, home humidifiers, showers and other fixtures that dispense tap water. Because contaminated aerosols can travel significant distances (2 km or more) and transmit disease (22, 23), it is difficult to conclusively identify the source for many LD cases (7). Among the various potential water sources of *Legionella*, CTs have been identified as a major source in multiple LD outbreaks (7, 24–27). Here, we focus on CTs due to their potentially important role in community outbreaks.

The first recognized LD outbreak occurred in Philadelphia in 1976, where 182 attendees of an American Legion convention at a hotel were infected and 29 of them died (28, 29). The source of *Legionella* was hypothesized to be the hotel’s air conditioning system but was not confirmed (7). Another LD outbreak occurred 2 years later at a hospital in Memphis, TN, and researchers were able to trace it back to the hospital’s air conditioning CT (30). During an investigation of an LD outbreak that occurred at a long-term care facility (LTCF) in North Carolina in September–October 2004, no evidence of *Legionella* colonization was found (25), either in the potable water or air-handling systems throughout the LTCF. However, the team isolated a common outbreak-causing *Lp* strain from an industrial CT located 0.4 km from the LTCF and recovered *Lp* DNA from the LTCF’s outdoor air-intake filters, indicating that aerosolized *Legionella* from the CT most likely entered the LTCF through the air-intake system or, possibly, through open windows. It was concluded (25) that residents of LTCFs can acquire LD from community sources, and a cluster of LD cases among LTCF residents does not necessarily indicate transmission from within the LTCF. An unusually long-lasting community-acquired outbreak of LD that occurred in northern Italy from 2005 to 2008 followed by a period of high LD incidence lasting up until 2012 was investigated (26). It was suggested that a hidden CT could have been the main cause of this uncommon long-lasting outbreak/incidence (this CT experienced an unintended shutdown in 2013 due to an economic crisis, and this coincided with a sudden decrease of LD incidence by 10-fold when compared to normal background levels). The work (26) highlights the difficulty in identifying the sources of LD incidence and the necessity of registration for all CTs. The largest LD outbreak in New York City (NYC) history occurred in July 2015, with 138 cases and 16 deaths (27). The initial epidemiological analysis did not find any common exposures (i.e. no common buildings visited by case patients), and case residences were spread across a 16.8 km^2^ area in the South Bronx. Based on whole genome sequencing and epidemiologic evidence obtained through a large-scale investigation that included sampling of 55 CTs in the area, a single CT located at the Opera House Hotel was implicated as the source of the outbreak (27). This historic LD outbreak spurred NYS to become the first large US jurisdiction to regulate the management of CTs to help prevent bacterial contamination. Ten NYCRR Part 4, Protection Against Legionella, was enacted in 2016 and requires the registration, inspection, maintenance, and annual certification of CTs.

**Fig. 2.**
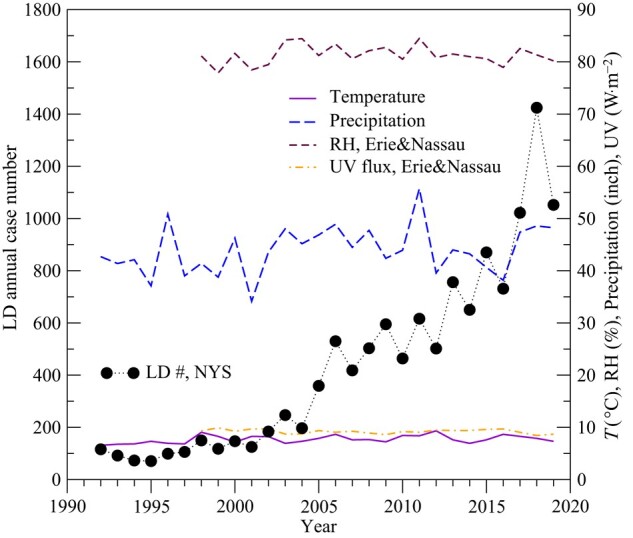
LD and influential environmental factors. Long-term trends of annual mean LD cases, temperature (*T*), RH, precipitation, and UV flux in NYS. RH and UV flux are the averaged at two sites (one in Erie County and the other in Nassau County in NYC, both in areas with high LD case number and IR), while *T* and precipitation are statewide averaged values of multiple sites.

The possible environmental factors influencing LD cases discussed in the literature include RH, *T*, precipitation, and UV radiation (7, 16–20) but the mechanisms and the magnitude of their influence remain unclear. While these factors may contribute to daily, seasonal, or even interannual variations of LD cases, they are not able to explain the observed long-term increasing trend of LD. We can see from Fig. 2 that there are no clear long-term trends of these factors in NYS. As pointed out in the Introduction section, the long-term increasing trend of LD remains unexplained in the LD research/health community and should be considered worrisome as we do not know if the increasing trend will continue (after the COVID-19 pandemic). The lack of understanding of the possible cause(s) makes it impossible to take preventative measures to mitigate the public health burden of LD.

**Fig. 3.**
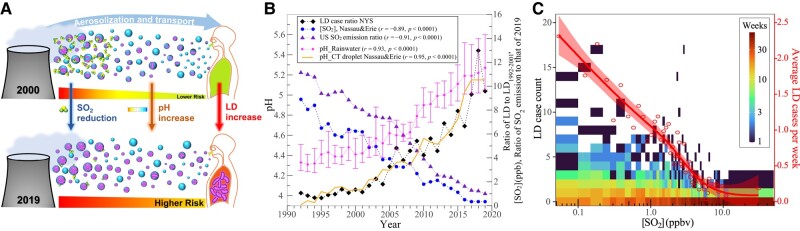
Declining SO_2_ associated with increasing LD. A) Schematic of the hypothesized mechanism of SO_2_ reductions affecting the pH of CT droplets and aerosols, survival rate of *Legionella*, and consequent impact on the risk of LD. B) Long-term trends (annual) of the ratio of LD cases with respect to its 1992–2001 mean, mean SO_2_ concentration ([SO_2_]) at two NYS sites (one in Erie County and the other in Nassau county in NYC), ratio of SO_2_ emission in the United States to that of 2019, mean pH of rainwater (measured) in NYS and CT droplet (calculated) at the two NYS sites (Erie and Nassau). In the calculation of CT droplet pH, mean [SO_2_] at the two sites is assumed. The error bars in rainwater pH curve show the SD. Pairwise Pearson’s correlation coefficients (*r*) and *P*-values are noted in the figure legend for long-term annual mean LD cases in NYS and four variables (SO_2_ concentration, SO_2_ emission, pH of rainwater, and pH of CT droplets). C) Binned scatterplot for weekly comparison of LD cases with 1-week-lagged [SO_2_] for these two sites (Erie and Nassau). Circles show average LD cases per week with respect to 1-week-lagged [SO_2_]. Curve shows generalized additive model fit and shading shows its 95% CI. Color scale (log_10_) indicates frequency of weeks. Bins are discretized nonlinearly along the *x*-axis such that the [SO_2_] distribution is uniform (in term of the number of weeks in each [SO_2_] bin).

In searching for the answers for the increasing LD trend as shown in Fig. 1, we have analyzed various factors that may potentially affect LD (including but not limited to those shown in Fig. 2) and found that SO_2_ concentration in the air has been decreasing in NYS and United States, in phase with and at a rate similar to that of the increasing LD during the last two decades. More importantly, we identified a physics-based mechanism/process that links SO_2_ reduction to LD increase. Figure 3A illustrates the process schematically and Fig. 3B and C provides observational data and a theoretical calculation supporting it. This process involves three key steps: (i) the water droplets emitted from CTs (and other water sources) uptakes SO_2_ from the ambient air, which decreases the droplet pH (i.e. more acidic); (ii) significant decrease in SO_2_ in the last two decades results in reduced SO_2_ uptake and thus lower acidity of the water droplets into a pH range (5.5–9.2) less stressful for *Legionella*; and (iii) viable *Legionella* in the contaminated droplet or aerosol from CT have longer lifetime at higher pH (i.e. low pH kills *Legionella* faster) and thus has higher concentration in the air, leading to increased LD in the last two decades. More details on the measurements and physics-based calculation supporting these steps are presented as follows.

#### Interaction of CT droplet with ambient air and change of its pH

The function of the CT is to dissipate away waste heat to the atmosphere. The wet or evaporative CT uses water spray to cool and emits a large amount of water droplets and vapor (31). The water droplets, sometime called drift or mist, generally evaporate quickly after exit. The evaporation time depends on the size of initial droplets, RH (near the CT exit and dependent on ambient RH, amount water vapor emitted, and mixing rate), and temperature. The initial size of droplets is generally in the range of 10-\!-50μm (larger than 50μm will fall to surface quickly) (32). Based on a droplet evaporation scheme that takes into account the evaporation energy balance and slightly lower temperature in the droplet surface (33), a droplet of 30μm takes ∼10–50 s to evaporate with an RH of 70–95% and at T=293K. Within this short time period, the droplet can take up a certain amount of SO_2_ (also H_2_O_2_) in the air and some of the SO_2_ is converted to sulfuric acid through aqueous chemistry oxidation by H_2_O_2_ and O_3_, similar to what occurs in clouds and rainwater (33, 34). As a result, the pH of the droplet decreases. Using an aqueous chemistry model (see supplementary material for more details) that considers CO_2_ equilibrium, SO_2_ uptake and oxidation (34, 35), we find that the pH of a pure water droplet in equilibrium with CO_2_ (390 ppmv) at T=293K is 5.6 and drops to 4.97, 4.58, 4.32, and 4.15 after 1, 10, 20, and 30 s of exposure, respectively, to ambient air with 5 ppb SO_2_, 2 ppb H_2_O_2_, and 30 ppb O_3_. Despite the short time scale, the SO_2_ uptake can substantially modify the pH of the CT droplets. It should be pointed out that the proposed mechanisms are not limited to CT droplets and can apply to other sources, such as hot tubs and fountains, from where *Lp*-contaminated water droplets are aerosolized.

#### Reduced SO_2_ results in increased pH of the *Legionella* contaminated water droplets

Ambient atmospheric SO_2_ concentration in the United States and especially NEUS have been reduced significantly, by around one order of magnitude from 2000 to 2019 (see https://www.epa.gov/air-trends/sulfur-dioxide-trends). The SO_2_ concentrations averaged from two Environmental Protection Agency (EPA) monitoring sites in NYS decreased from ∼8 ppb in early 1990s to ∼5 ppb in early 2000s and then to 0.5 ppb in later 2010s (filled blue circles in Fig. 3B). Based on the SO_2_ uptake and aqueous chemistry model (34, 35), these decreases in SO_2_ concentrations in the air reduce the amount of SO_2_ taken up and oxidized into sulfate and thus increase the pH of CT droplets, from <∼4.1 before 2002 to ∼5.1 in 2019 (yellow line in Fig. 3B). The model calculated pH increase is consistent with the measured change of rainwater pH during the period (pink symbols). The pH of rainwater is higher than that of CT droplets and is as expected because of the large volume of rainwater (thus more diluted acid) and the limitation of SO_2_ available for uptake during rain events. It should be noted that the pH change of CT droplets shown in Fig. 3B does not consider the uptake of HNO_3_ and NH_3_ or particles in the air, which also exhibit a long-term trend. HNO_3_ acts to decrease, while NH_3_ increases pH. The pH of particles in the air has a long-term increasing trend due to SO_2_ emission reduction. While the effect of interactions with HNO_3_, NH_3_, and particles remains to be investigated, it is expected to enhance the increasing trend of CT droplet pH as shown in Fig. 3B and thus the validity of this process.

The long-term trend results of Fig. 3B and the strong correlations (*r*) between LD cases and the four variables—SO_2_ concentration (−0.89,P≪0.001), SO_2_ emission (−0.91,P≪0.001), pH of rainwater (0.93,P≪0.001), and pH of CT droplets (0.95,P≪0.001)—are based on annual mean values and one may question the temporal mismatch of annual mean LD cases and SO_2_ concentrations. To address this, Fig. 3C shows the variability of CDC-reported LD cases (1992–2019) with 1-week-lagged [SO_2_] in Erie and Nassau counties. Sensitivity analysis indicates the LD event to be most sensitive to the 1-week-lagged [SO_2_] and is consistent with the period from exposure to symptom onset (36). Occurrence of large numbers of LD cases typically occurs after weeks of low [SO_2_]. The average LD cases per week increase with decreasing SO_2_, with a maximum of 2.01 LD cases per week when [SO_2_] is lowest (0–0.25 ppbv). So that this analysis is unaffected by the statistical properties of the [SO_2_] distribution (right-skewed), bins are discretized such that the [SO_2_] distribution is uniform. Additionally, randomization of [SO_2_] breaks its association with LD cases (Fig. S1). The analysis is also conducted for three other diseases (rationale for selection is discussed in detail in the Materials and methods section), for which this relationship with [SO_2_] is not observed (Fig. S2). This temporally fine-grained (weekly and lagged) analysis further supports the hypothesized role of [SO_2_] reductions on LD case incidence observed for the annual scale in Fig. 3B.

#### *Legionella* survive longer in lower [SO_2_] and lead to more LD cases

Water is the natural habitat for *Legionella*, which can survive for more than a year in tap water (37). One of the most significant environmental parameters influencing the growth and survival of microbes (including *Legionella*) is the local concentration of protons (hydrogen ions, H^+^), which is measured as pH $\left(=-\log_{10}\;(\alpha_{H^{+}}\right)$, where $\alpha_{H^{+}}$ is the hydrogen ion activity in a solution) (38, 39). At low pH, the protonation of biological molecules adversely affects their charge, structure, and function (39). The survival of *Legionella* has been observed to be sensitive to pH (40, 41). It was shown (40) that *Legionella* survived exposure to hydrochloric acid for 30 min at pH of 2.0, but exposure for 1 min in pH 1.7 was completely inhibitory. In the laboratory measurements examining the effect of drying, pH, and heat on the survival of *Legionella*, it was shown (41) that drying has most significant impact and pH also has a strong effect. Only 0.01 to 0.1% of *Legionella* survived after the first 30 s of drying but these remaining (viable) *Legionella* can survive up to ∼90 min. This time scale of drying and surviving is consistent with LD outbreaks generally being limited to ∼10 km of the contaminated CT, roughly within ∼60 min of the source assuming a mean wind speed of ∼3 m s^−1^. With regard to the effect of pH, the percentages of *Legionella* in tap water remaining viable after 24 h at pH of 6, 5, and 4 are 88, 80, and 12%, respectively (41). Also, *Legionella* were only able to survive for a few minutes at pH of 2 to 3. The measured strong sensitivity of *Legionella* survival rate to pH when pH <∼5 offers a possible explanation for the increase of LD cases in NYS and United States as shown in Figs. 1 and 3B. It should be noted that the calculated CT droplet pH values given in Fig. 3B are for average conditions and in reality some droplets may have a much lower pH if exposed to [SO_2_] much higher than the average [SO_2_]. In addition, as CT droplets evaporate, acid in the droplets becomes more concentrated and acidity increases. We would like to reemphasize that pH of CT droplets shown in Fig. 3B are subject to variations depending on drying time and interactions with species other than SO_2_ but the trend shall hold. Also, the pH of aerosolized CT droplets is expected to be higher but the trend should hold, i.e. pH of both CT droplets and aerosols has been increasing in the last two decades. We believe that this increasing pH of CT droplet/aerosols (Fig. 3B) combined with the observed (40, 41) dependency of *Legionella* survivability on pH may have contributed to the rapid increase of LD in the United States in recent years.

#### Statistical analysis further supporting the proposed mechanism associating [SO_2_] declines with LD increase

**Fig. 4.**
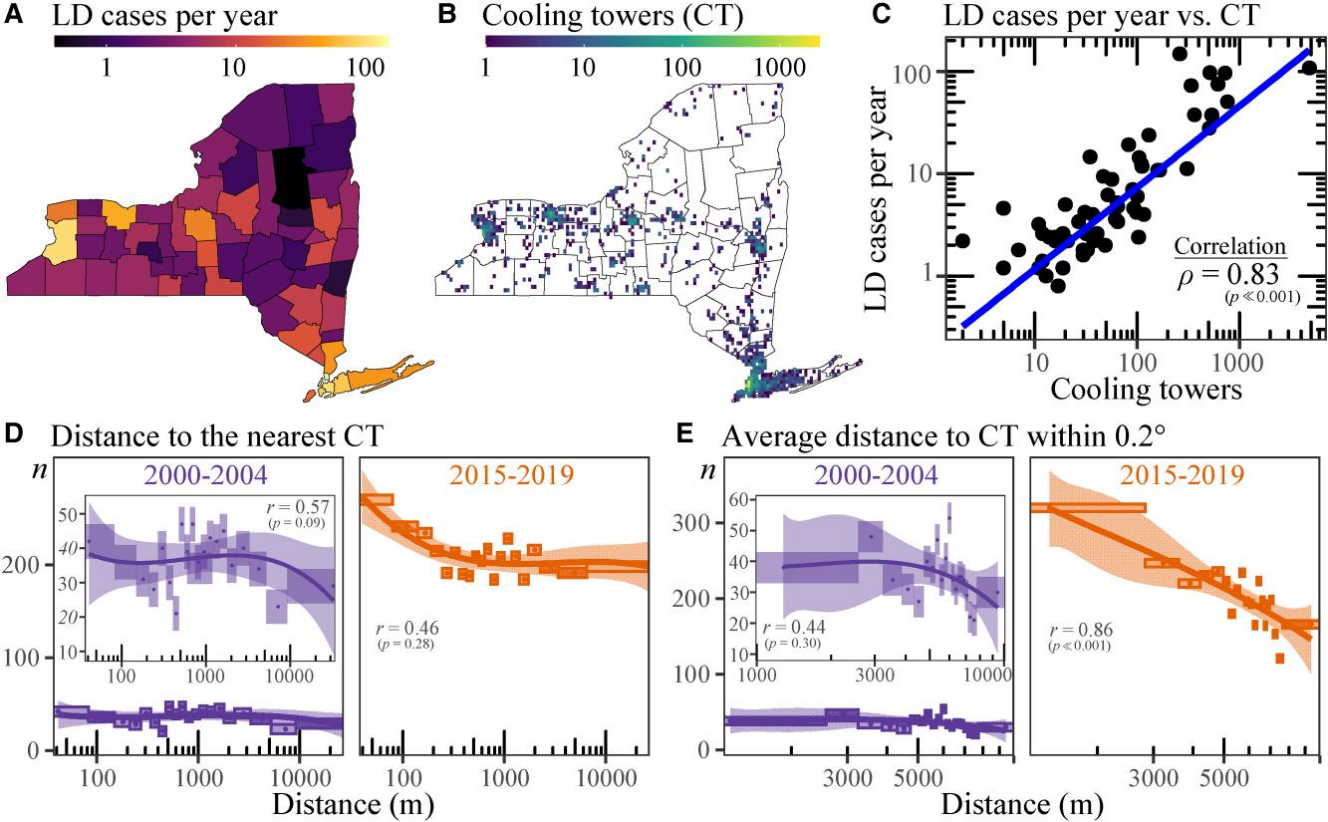
LD and CTs in NYS. A) County-level LD cases per year during the period 2015–2019. B) CT locations and counts based on NYS registered CTs. CTs are binned into $0.05^{\circ }\times 0.05^{\circ }$ boxes with color scale (log_10_) showing the number per bin. C) Scatter plot of county-level LD cases per year (2015–2019 average case per year) vs. CT counts. Number of LD hospitalizations (*n*) vs. D) distance from the nearest CT and E) average distance to CT within $0.2^{\circ }$. The *x*-axis marks distance on a logarithmic scale. Due to the lower values during 2000–2004, the insets zoom in on the *y*-axis. Curve indicates the cubic polynomial fitting and shading indicates its 95% CI. To account for population number, the distance (*x*-axis) is discretized per ventile of acute appendicitis hospitalizations as described in text.

The strong correlation in spatial distribution of LD cases and CTs in NYS, as we show in Fig. 4A–C, indicates that CTs satisfy the necessary condition to be a major source of LD. One limitation of this colocation analysis is the distribution of population density (and hence LD cases) that may also correlate with CT counts. To resolve this and to confirm our proposed physics-based mechanism of increasing LD cases, we analyzed the number of LD hospitalizations vs. distance from the nearest CT during the two 5-year periods (Fig. 4D and E), using the unique address-identified LD hospitalizations from the Statewide Planning and Research Cooperative System (SPARCS) and CT data available in NYS. Figure 4D shows the variation of LD hospitalizations with respect to distance of residence to the nearest CT.

With linearly increasing distance from a CT, effects on the population number are encompassed: increases due to the increasing annular area and decreases due to reduced probability of not being near a CT (Fig. S3). It is important to account for this potentially confounding effect of population density, ideally by comparing LD hospitalizations to the remainder (unaffected) population with CT distance. Unfortunately, such an individual-level dataset for every NYS resident is unavailable, so we use an alternative approach. Acute appendicitis results from a blockage in the lining of the appendix that triggers inflammation; its incidence in a population is random and should have no link with CTs. Thus, normalizing by the distribution of appendicitis hospitalization, as a representative sample of the population, should eliminate the effects of population density. For this, the *x*-axis in Fig. 4D is normalized by distance ventiles (20 equal frequency bins) for hospitalizations due to acute appendicitis. If there were also no effect of CT location on LD hospitalization incidence, we would observe a uniform distribution. However, Fig. 4D shows that the number of LD hospitalizations are greater than expected for residents closer to the CT. Additionally, the increased LD hospitalizations between 2000–2004 and 2015–2019 show a greater rate of increase closer to the CT. Further sensitivity analyses are presented (Fig. S4) using ischemic heart disease (reflecting random affliction in the population and additionally shares LD risk factors) and streptococcal pneumonia (pneumonia caused by a different bacteria: *Streptococcus pneumoniae* and with a different transmission mechanism). Fitting a cubic polynomial permits analysis of when there is deviation from a uniform distribution. The point of maximum curvature (42) can be used to determine the effective range of a CT, defined as the distance from a CT up to which there are more LD hospitalizations than expected. In Fig. 4D, these cubic polynomial fits are not statistically significant for 2000–2004 (r=0.57,P=0.09) and 2015–2019 (r=0.46,P=0.28) to quantitatively determine the effective CT range, despite the visually evident increase in LD cases as distance to the CT reduces. However, the fit for 2015–2019 becomes statistically significant (r=0.66,P=0.04) when not considering distances >6.5km and shows an approximate effective range of at least ∼1.2 km (lower bound of 95% CI). It should be noted that Fig. 4D shows the distance of the nearest CT (to the residence address of the reported LD hospitalization) and in reality, a person can be affected by CTs other than the nearest one closest to their residence. Figure 4E examines the sensitivity to this using the mean distance to all CTs within $0.2^{\circ }$ (assuming a CT effective range of <∼20 km). As in Fig. 4D, for 2000–2004, there is no statistically significant fit (r=0.44,P=0.30) to quantify the average effective CT range from the observed increase in LD cases as average distance to CTs decline. For 2015–2019, the fit (r=0.86,P≪0.01) permits estimation of the average effective CT range. Up to an average CT distance of ∼7.3 km (lower bound of 95% CI), a statistically significant and higher risk of LD hospitalization exists and increases as the average distance to a CT reduces. The earlier implicit assumption that emission of *Lp*-contaminated CT droplets is random (among CTs) is justified by the consistency between Fig. 4D and E and they fully support the proposed increased survival time and hence areas affected by *Lp* from CTs under conditions of lower [SO_2_] (Fig. 3B and C).

## Discussion

*Legionella* pose significant risk to the health of vulnerable people when water containing the bacteria becomes aerosolized. Motivated by the tripling of reported LD cases in the United States from 2001 to 2012, policies and guidelines for the primary prevention of LD were examined and it was concluded that the disease deserves a higher priority for public health policy development and research (43). Barely 6 years later, in 2018, reported LD cases in the United States had almost tripled further. Although the COVID-19 pandemic has significantly affected the reported LD cases since 2019, it is important to assess and prepare for the LD incidence postpandemic. In this regard, it is vital to understand the reasons behind the rapid increase in LD cases in the United States since 2001.

Here, our analysis suggests that factors known to affect LD cannot explain its long-term trend and we further demonstrate that declining sulfur dioxide air pollution results in lower aerosol acidity, which is associated with the increase in LD, potentially via prolonged survival of *Legionella*. Our estimation of aerosol acidity is limited by the paucity, due to measurement challenges (44), of direct measurements of ambient atmospheric aerosol pH. In addition, we currently do not consider other atmospheric species capable (minor, however, compared to SO_2_) of affecting aerosol acidity. The current finding has implications for forecasting LD risks and needed interventions as SO_2_ concentration changes are expected to continue. While this work highlights the need to holistically consider the complexities of SO_2_ concentration changes, it would be unwise to argue against its reductions, which has well-recognized health benefits. Public health officials and clinicians should be aware of the potentially increased risk for legionellosis during periods of high CT use and low SO_2_ concentrations, targeting at-risk populations in areas of chronically high incidence. Knowledge of this risk may improve diagnostic and treatment decisions surrounding cases of community-acquired pneumonia, of which *Legionella* are increasingly recognized as a cause of. Environmental studies designed to evaluate the precise effect of SO_2_ concentrations (and other relevant species) on *Legionella* growth, spread, and acquisition, along with systematic laboratory measurements of the impact of pH on *Legionella* survivability in drying water droplets, are difficult but necessary to better understand the association we uncovered and its dependence on various factors. Identification of the specific mechanisms and events that lead to increased transmission may lead to opportunities for prevention of sporadic LD and reduction of *Legionella* exposure disparity in underserved communities.

## Materials and methods

### Calculation of CT droplet pH

The pH of CT droplets was calculated using the SO_2_ uptake and aqueous chemistry model (34, 35). For the results shown in Fig. 3B, annual mean SO_2_ concentrations at the two sites were used, with a fixed temperature of 290 K. CO_2_, O_3_, and H_2_O_2_ mixing ratios were assumed to 390 ppmv, 30 ppbv, and 2 ppbv, respectively. The pH calculation, started with a droplet in equilibrium with CO_2_, was based on the uptake of SO_2_ and its oxidation during a period of 30 s.

### Sensitivity analyses

Sensitivity of the [SO_2_]-disease association to the [SO_2_] distribution is examined, as it is right-skewed with typical values between 0 and 5 ppbv. It may be that since low [SO_2_] values are more frequent, the occurrence of cases is more probable during low [SO_2_]. To account for this potential effect, a nonlinear binning of [SO_2_] is carried out such that it is uniformly distributed. To rule out any remainder effects of the statistical properties of the [SO_2_] distribution impacting the association we examine, for comparison, we randomize [SO_2_] such that its statistical distribution is the same, but any hypothesized effect on LD cases is broken.

Sensitivity of the [SO_2_]-disease association to the CDC data reporting quality is examined using the SPARCS data for Legionellosis hospitalizations (International Classification of Diseases, Tenth Revision (ICD-10) codes “A481” and “A482” and International Classification of Diseases, Ninth Revision (ICD-9) codes “48284” and “04089”). This provides additional corroborative analysis with a secondary dataset and leverages its superior quality of reporting despite temporally shorter data coverage.

Sensitivity of the [SO_2_]-disease association to the disease is examined using three reference diseases: acute appendicitis (ICD-10: “K35” and ICD-9: “540”), streptococcal pneumonia (ICD-10: “J13” and ICD-9: “481”), and chronic ischemic heart disease (ICD-10 category code “I25” and corresponding relevant ICD-9 codes in the “41” and “42” categories).

Sensitivity of the CT-disease association to population changes occurring at different distances from a CT is examined. In an ideal analysis, one would compare LD hospitalizations to the remainder (unaffected) population with CT distance. Unfortunately, the required individual-level dataset for all of the population is unavailable. This necessitates an alternative to appropriately scale the distance to the nearest CT to minimize effects of population change. Three candidate diseases—acute appendicitis, chronic ischemic heart disease, and streptococcal pneumonia—are selected with the following rationale: if there is no individual-level population data, a representative sample of such should suffice. Acute appendicitis results from a blockage in the lining of the appendix that triggers inflammation; it may be assumed that its occurrence in the population is random with respect to cooling towers, and therefore normalizing by the spatial distribution of appendicitis should eliminate the effects of population density. However, it may be unsuitable for other individual-level characteristics. For instance, appendicitis typically affects the young (SPARCS geometric median age is 27.3 years) and is not expected to reflect the risk factors for LD (median age of 60.0 years). As a reminder, those most vulnerable to LD are male, over 50 years of age, have a history of smoking, have chronic respiratory diseases, diabetes, are immunocompromised, and/or minorities. Regardless, to eliminate only the population density effects, hospitalizations due to acute appendicitis is a suitable reference. Chronic ischemic heart disease typically results from narrowed heart arteries; it may be assumed that its occurrence in the population is random with respect to CTs. Additionally, this disease (median age of 65.0 years) and typical comorbidities may better reflect both the effects of population density around CTs and share elevated-risk factors for LD. For additional reference, we also choose a disease with similar presentation as LD but different etiology and transmission mechanism: streptococcal pneumonia.

Sensitivity of the CT-disease association to the implicit assumption that the probability of a CT being contaminated with *Lp* is random, due to consideration of only the nearest CT, is examined. We consider the average distance to all CTs within $0.2^{\circ }$. The $0.2^{\circ }$ upper bound is chosen to consider the extremely low probability of a CT beyond ∼20 km having any impact on an individual.

### Statistical information

Pearson’s correlation coefficients were calculated for long-term annual mean LD cases in NYS and four variables (SO_2_ concentration, SO_2_ emission, pH of rainwater, and pH of CT droplets). Spearman’s rank correlation coefficient is used to quantify the correlation between LD cases per year and CT counts.

LD incidence was calculated by dividing the number of reported LD cases by the jurisdiction population estimate and then multiplied by 100,000. Adjusted distance was calculated by selecting ventiles for distance to CTs for a reference disease assuming no effect of CTs. Corresponding to these distances, LD cases were binned. If there is no effect of CTs on LD, it is expected to be as uniformly distributed as ischemic heart disease.

## Supplementary Material

pgae085_Supplementary_Data

## Data Availability

Demographic data from 1992 to 2019 are publicly available and were obtained from the US Census Bureau (45–47) at the state level and for NYS at the county level from New York State Department of Labor (48). All CTs in New York State are required to be registered and their information is made publicly available by New York State Department of Health (49). US county-level LD case data were provided by the US CDC. Patient-level health data were obtained from the NYS comprehensive all payer data reporting system: SPARCS. Identifiable individual-level health data are not openly available and can be requested from the Office of Quality and Patient Safety, New York State Department of Health. The publicly available SO_2_ concentration data are from EPA Air Data, SO_2_ emission data from EPA air emissions inventories, UV and RH from National Solar Radiation Database, precipitation and temperature data from NOAA National Centers for Environmental Information, and rainwater pH from National Atmospheric Deposition Program. Data and code for the analyses presented here are available at https://doi.org/10.6084/m9.figshare.25157852.
