# Supplementary material for: Mysteriously rapid rise in Legionnaires’ disease incidence correlates with declining atmospheric sulfur dioxide
Source: PNAS Nexus. 2024 Mar 12;3(3):pgae085. doi: 10.1093/pnasnexus/pgae085 (PMC10929586; doi:10.1093/pnasnexus/pgae085)
Supplement: pgae085_Supplementary_Data [file pgae085_supplementary_data.pdf]

PNAS nexus

# Supporting Information for

## Mysteriously rapid rise in Legionnaires' disease incidence correlates with declining atmospheric sulfur dioxide

**Fangqun Yu[a,1,2], Arshad Arjunan Nair[a,1,2], Ursula Lauper[b], Gan Luo[a], Jason Herb[a], Matthew Morse[b], Braden Savage[b], Martin Zartarian[b], Meng Wang[c], and Shao Lin[d]**

[a]Atmospheric Sciences Research Center, University at Albany, State University of New York, Albany, NY 12226; [b]Bureau of Water Supply Protection, New York State Department of Health, Albany, NY 12223; [c]School of Public Health and Health Professions, University at Buffalo, State University of New York, Buffalo, NY 14214; [d]School of Public Health, University at Albany, State University of New York, Albany, NY 12144

[1]F.Y. and A.A.N. contributed equally to this work; [2]To whom correspondence should be addressed. **E-mail:** fyu@albany.edu & aanair@albany.edu.

### This PDF file includes:

Supporting text
Figs. S1 to S5
SI References

## Supporting Information Text

**Aqueous chemistry model to calculate $SO_2$ uptake and pH of a cooling tower (CT) water droplet.** Similar to those in clouds or fog, the uptake of sulfur dioxide ($SO_2$) by CT droplets involves several chemical reactions that occur in the aqueous phase. When $SO_2$ comes into contact with CT droplets, it can undergo dissolution and subsequent reactions, leading to the formation of sulfate and decrease of pH values of the droplet. The $SO_2$ aqueous chemistry process has been well described in textbooks on atmospheric chemistry (1, 2) and has been implemented in various models of atmospheric chemistry. In this study, we use the $SO_2$ aqueous chemistry module (3–5) of the widely-used community atmospheric chemistry model GEOS-Chem (6). The main processes are briefly summarized here.

1. Dissolution: $SO_2$ dissolves in the water content of cloud droplets to form aqueous sulfur dioxide ($SO_2$(aq)).

2. Hydration: $SO_2$ (aq) can undergo hydration reactions, forming bisulfite ions ($HSO_3{}^-$) and sulfite ions ($SO_3{}^{2-}$):

   (R1a) $SO_2\,(aq) + H_2O \rightleftharpoons HSO_3{}^- + H^+$

   (R1b) $HSO_3{}^- \rightleftharpoons SO_3{}^{2-} + H^+$

3. Oxidation by dissolved $H_2O_2$:

   (R2a) $HSO_3{}^- + H_2O_2 \rightleftharpoons SO_3OOH^- + H_2O$

   (R2b) $SO_3OOH^- + H^+ \rightleftharpoons H_2SO_4$

4. Oxidation by dissolved $O_3$:

   (R3) $SO_3{}^{2-} + O_3 \rightleftharpoons SO_4{}^{2-} + O_2$

The oxidation by dissolved $H_2O_2$ (R2) is independent of pH while that by dissolved $O_3$ (R3) is significant only at pH $> 6$. Sulfur in the aqueous phase can also be oxidized by other less important oxidants (such as $O_2$, $NO_2$, $NO_3$, etc.). In this work, we only consider the aqueous phase oxidation of $SO_2$ by $H_2O_2$ and $O_3$.

We calculate cloud water pH iteratively by using the concentrations of sulfate, total ammonium (ammonium + ammonia), total nitrate (nitrate + nitric acid), $SO_2$, and $CO_2$ based on their effective Henry's law coefficients and cloud liquid water content (7, 8), with the iterative calculation updated to use Newton's method (9). The initial pH of droplets is assumed to be that of pure water in equilibrium with $CO_2$ and is the same for all case years (with different $SO_2$ concentrations). After about 30 s of exposure to $SO_2$ at concentrations typical of those in New York State during the last two decades, the pH of CT droplets is dominated by the $SO_2$ taken up and is insensitive to initial pH concentrations.

**Breaking the association between [$SO_2$] and LD cases.** In Fig. 3*C* (main text), the opposite relationship between LD cases per week and 1-week-lagged [$SO_2$] was demonstrated. The analysis is repeated on randomized [$SO_2$] data (Fig. S1*A*). No association between [$SO_2$] and LD case count exists in this case. Additionally, the same analysis (as in Fig. 3C; main text) is conducted for the SPARCS-reported legionellosis hospitalizations (Fig. S2*A*) and with the randomization of [$SO_2$] (Fig. S1*B*). We use the ICD-10 codes "A481" & "A482" and ICD-9 codes "48284" & "04089" to identify legionellosis hospitalizations. These sensitivity analyses confirm that the properties of the [$SO_2$] distribution do not affect the relationship obtained in Fig. 3*C* (main text).

**[$SO_2$] and cases of LD and reference diseases.** For further sensitivity analyses (Fig. S2), we use SPARCS-reported hospitalizations to compare the [$SO_2$]–disease relationship found for Legionnaire's disease (Fig. S2*A*) with that for three other diseases: acute appendicitis (Fig. S2*B*), streptococcal pneumonia (Fig. S2*C*), and chronic ischemic heart disease (Fig. S2*D*). We find no such relationship for these three diseases, in fact the opposite relationship (increasing $SO_2$ and increasing hospitalizations) is seen for ischemic heart disease and pneumonia (in line with literature), further confirming the solidity of our findings in Fig. 3*C*.

**Distance to nearest cooling tower and cases of LD and reference diseases.** In Fig. 4*D* (main text), we summarize the relationship between LD hospitalizations and distance to the nearest cooling tower. Figure S3 demonstrates this for LD (Fig. S3*A*) and the three other diseases (Fig. S3*B*–*D*) without any normalization of the distance. For LD, hospitalizations increase from 2000–2004 to 2015–2019, unlike for the other diseases. Additionally, the median distance to the nearest CT (denoted by dashed vertical line segment) becomes smaller with time. This indicates that more of the increase from 2000–2004 to 2015–2019 occurs closer to the nearest CT, consistent with the observation in Fig. 4*D* (main text) that the rate of increase of LD hospitalizations is greater closer to the nearest CT. At first glance, it may seem as though there are low number of cases closer to the cooling tower, with peak values around 600–900 m. This is due to the inappropriate discretization of the distance, which captures the contrasting effects of larger distances from a CT covering larger annular areas and thus larger populations (or its diseased subsets) versus the reducing probability of being far away from a cooling tower.

Adjusting for this effect, the analysis in Fig. 4*D* (main text) is extended in Fig. S4. The first and last panels of the top row of Fig. S4 is equivalent to Fig. 4*D* (main text), with quintiles for acute appendicitis hospitalizations with distance to the nearest cooling tower being used to bin LD hospitalizations. The same is shown using quintiles for chronic ischemic heart disease (center row) and quintiles for streptococcal pneumonia (bottom row). This sensitivity analysis shows that the finding for LD hospitalization relationship with cooling tower location is consistent. Non-flattening of the curve at large distance for LD with respect to pneumonia is speculated to be due to the lower transmission probability in remote areas (which have the nearest cooling tower farther away). In any case, streptococcal pneumonia is not an ideal reference as described earlier.

**Average cooling tower distance and cases of LD and reference diseases.** The analysis in Fig. S4 is repeated for the average, in lieu of nearest, distance to the cooling towers. The first and last panels of the top row of Fig. S5 is equivalent to Fig. 4*E* (main text). We find the observed relationships: (a) increasing LD hospitalizations with time, (b) largest increases closer to cooling tower, and (c) increasing effective range of cooling tower remain consistent.

## SI References


1. JH Seinfeld, SN Pandis, *Atmospheric Chemistry and Physics.* (Wiley-Blackwell, Hoboken, NJ), 3 edition, (2016).
2. MZ Jacobson, *Fundamentals of Atmospheric Modeling.* (Cambridge University Press), (2005).
3. DJ Jacob, Chemistry of OH in remote clouds and its role in the production of formic acid and peroxymonosulfate. *J Geophys. Res* **91**, 9807 (1986).
4. H Liu, DJ Jacob, I Bey, RM Yantosca, Constraints from 210pb and 7be on wet deposition and transport in a global three-dimensional chemical tracer model driven by assimilated meteorological fields. *J. Geophys. Res. Atmospheres* **106**, 12109–12128 (2001).
5. B Alexander, et al., Sulfate formation in sea-salt aerosols: Constraints from oxygen isotopes. *J. Geophys. Res. Atmospheres* **110** (2005).
6. I Bey, et al., Global modeling of tropospheric chemistry with assimilated meteorology: Model description and evaluation. *J. Geophys. Res. Atmospheres* **106**, 23073–23095 (2001).
7. B Alexander, et al., Isotopic constraints on the formation pathways of sulfate aerosol in the marine boundary layer of the subtropical northeast atlantic ocean. *J. Geophys. Res. Atmospheres* **117** (2012).
8. G Luo, F Yu, JM Moch, Further improvement of wet process treatments in geos-chem v12.6.0: impact on global distributions of aerosols and aerosol precursors. *Geosci. Model. Dev.* **13**, 2879–2903 (2020).
9. JM Moch, et al., Global importance of hydroxymethanesulfonate in ambient particulate matter: Implications for air quality. *J. Geophys. Res. Atmospheres* **125** (2020).

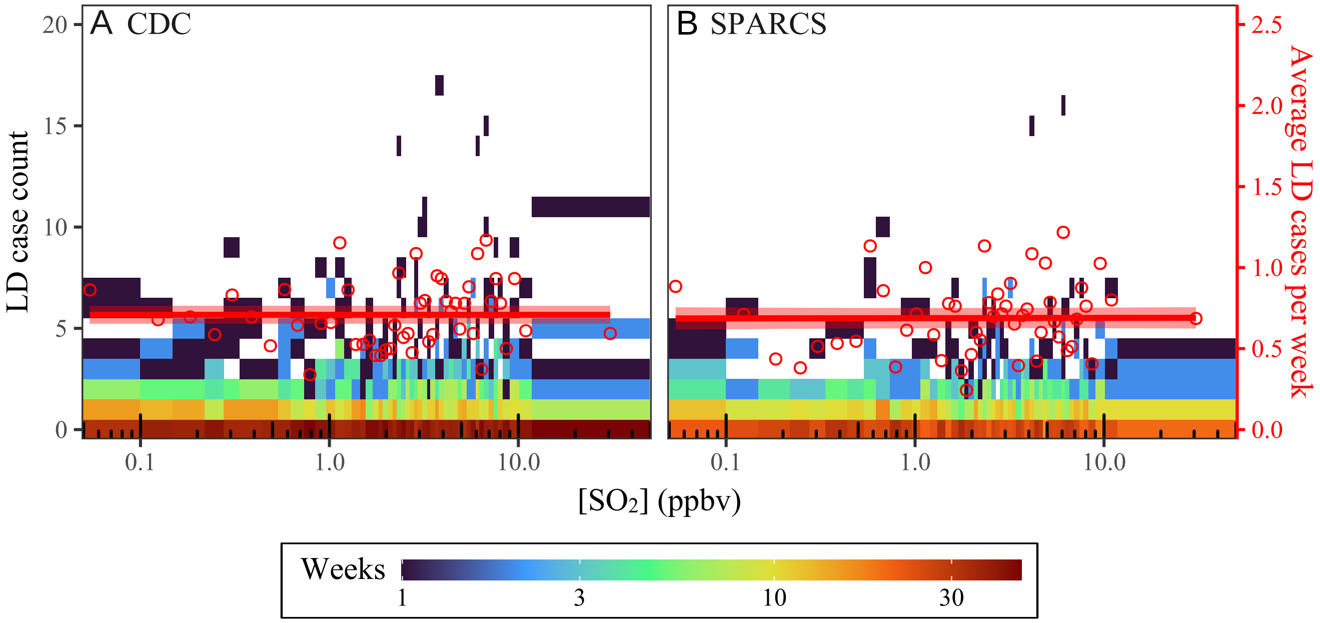


**Fig. S1.** Association between [$SO_2$] and Legionnaires' disease. (*cf.* Fig. 3*C*) Binned scatterplot for weekly comparison of *(A)* CDC-reported LD cases and *(B)* SPARCS-reported LD hospitalizations with 1-week-lagged randomized [$SO_2$] for Erie and Nassau. Red circles show average LD cases per week with respect to 1-week-lagged [$SO_2$]. Red curve shows generalized additive model fit and red shading shows its 95% confidence interval. Color scale ($\log_{10}$) indicates frequency of weeks. Bins are discretized non-linearly along the x-axis such that the [$SO_2$] distribution is uniform.

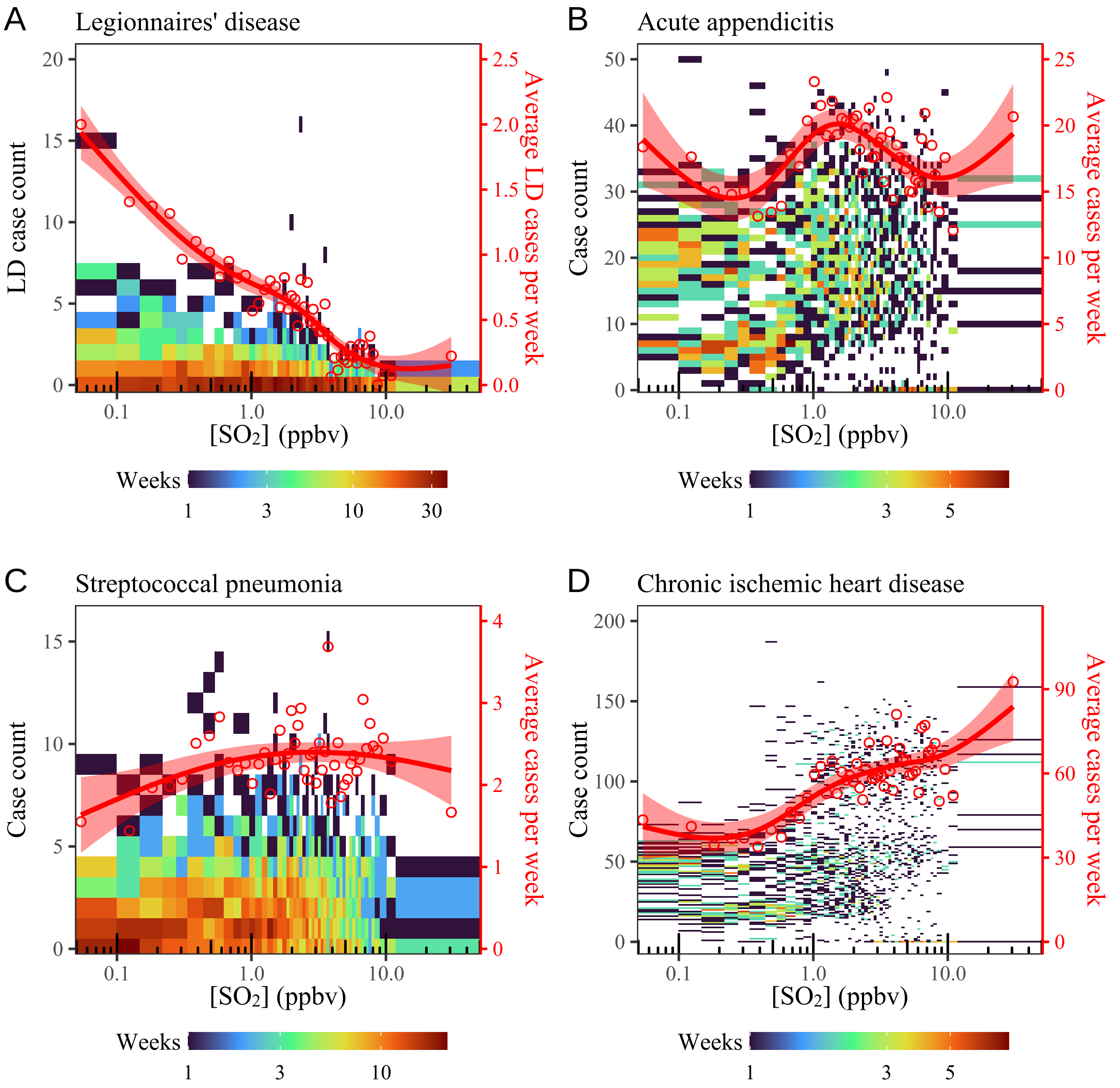


**Fig. S2.** $[SO_2]$–disease associations. (*cf.* Fig. 3*C*) Binned scatterplot for weekly comparison of LD hospitalizations (SPARCS; 2000–2019) with 1-week-lagged $[SO_2]$ combined for Erie and Nassau. Plot is faceted by: *(A)* Legionnaires' disease, *(B)* acute appendicitis, *(C)* streptococcal pneumonia, and *(D)* chronic ischemic heart disease. Red circles show average hospitalizations per week with respect to 1-week-lagged $[SO_2]$. Red curve shows the generalized additive model fit and red shading shows its 95% confidence interval. Color scale ($\log_{10}$) indicates frequency of weeks. Bins are discretized non-linearly along the x-axis such that the $[SO_2]$ distribution is uniform.

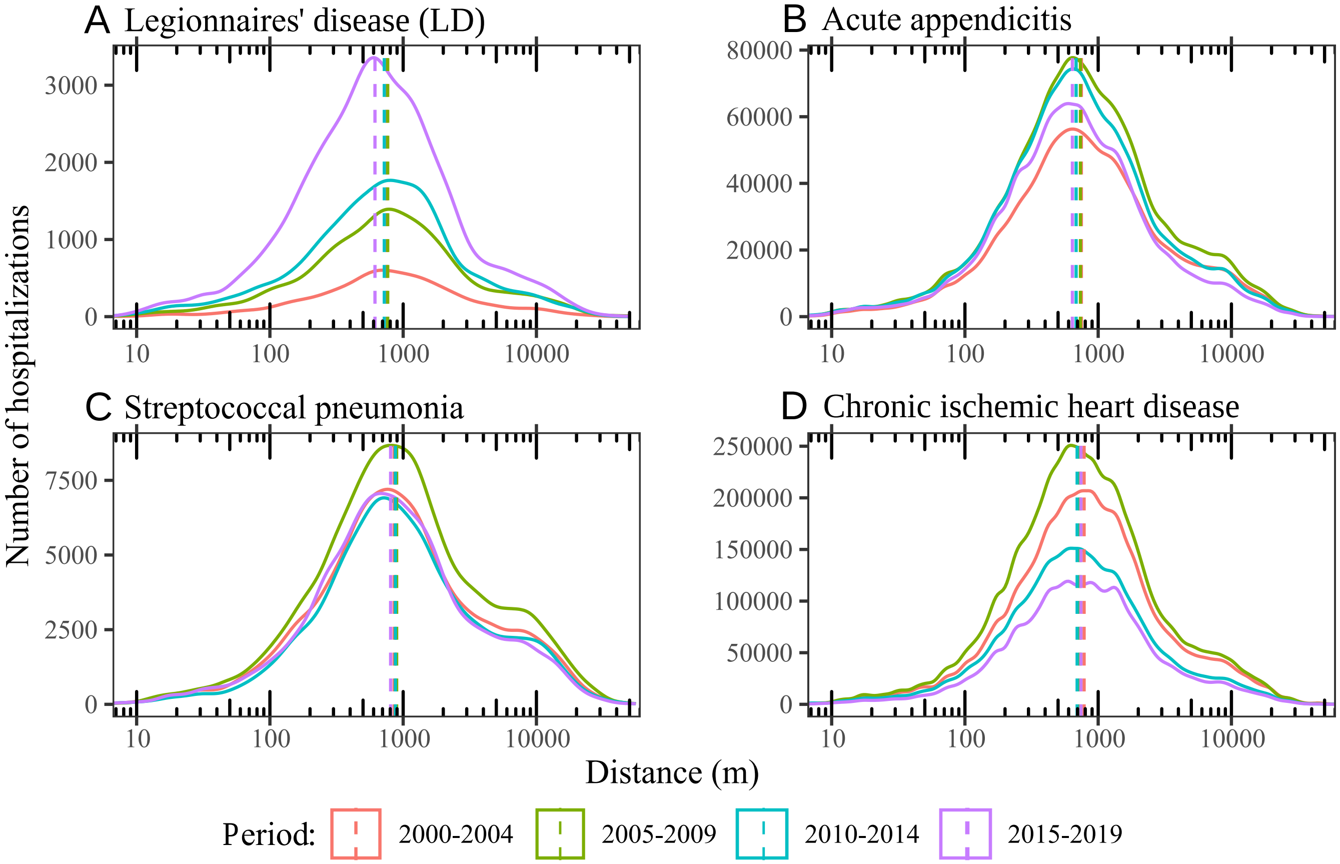


**Fig. S3.** Spatial relationship between Legionellosis and cooling towers. Kernel density estimate for the number of hospitalizations with respect to distance from the nearest CT. Plot is faceted by: *(A)* Legionnaires' disease, *(B)* acute appendicitis, *(C)* streptococcal pneumonia, and *(D)* chronic ischemic heart disease. Colors indicate the four five-year periods: 2000–2004 (red), 2005–2009 (green), 2010–2014 (blue), and 2015–2019 (purple).

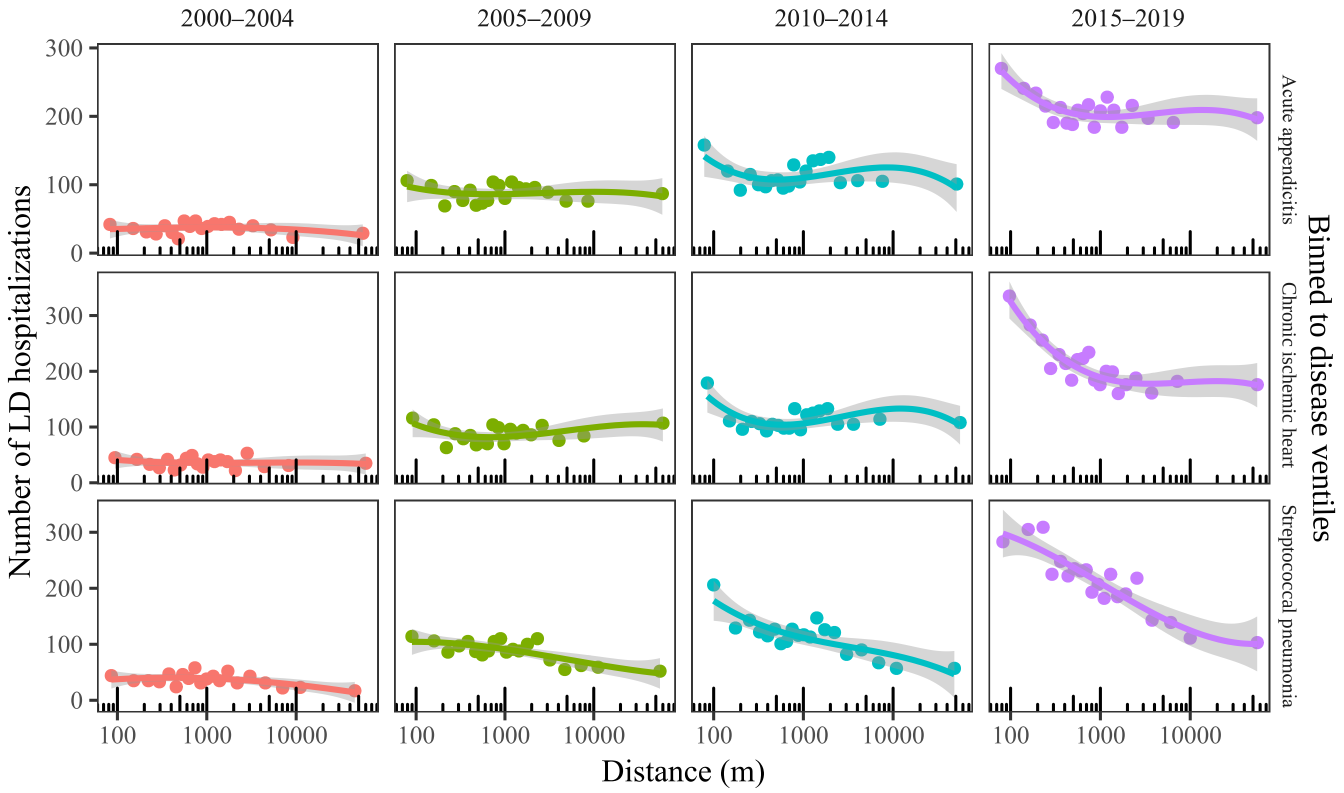


**Fig. S4.** Legionellosis hospitalizations and the nearest cooling tower. Number of LD hospitalizations versus distance from the nearest CT. The distance (x-axis) is discretized per ventile of (*top row*) acute appendicitis, (*center row*) chronic ischemic heart disease, and (*bottom row*) streptococcal pneumonia. Curve indicates the cubic polynomial fitting and shading indicates its 95% confidence interval. Plots are faceted column-wise by the four five-year periods: 2000–2004 (red), 2005–2009 (green), 2010–2014 (blue), and 2015–2019 (purple).

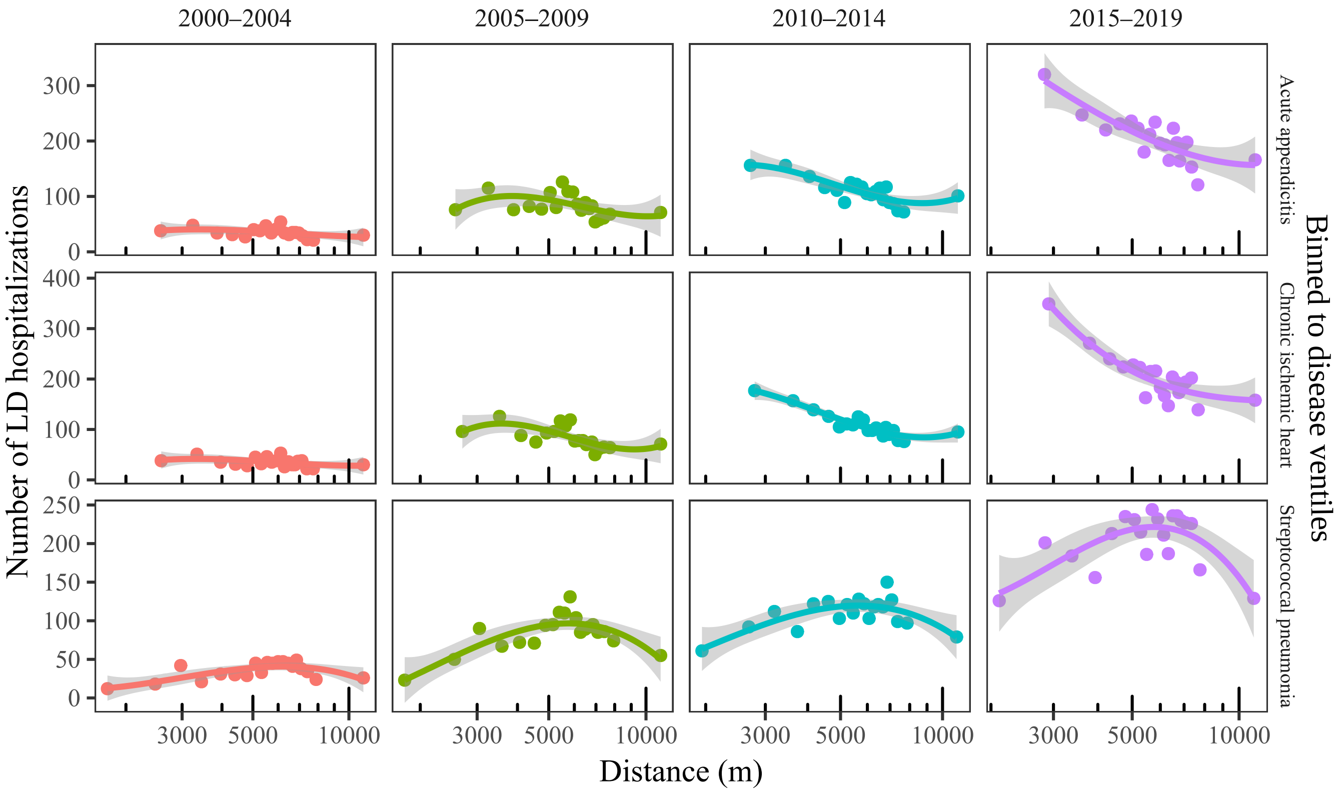


**Fig. S5.** Legionellosis hospitalizations and average cooling tower distance. Number of LD hospitalizations versus average distance from CTs (within 0.2°). The distance (x-axis) is discretized per ventile of (*top row*) acute appendicitis, (*center row*) chronic ischemic heart disease, and (*bottom row*) streptococcal pneumonia. Curve indicates the cubic polynomial fitting and shading indicates its 95% confidence interval. Plots are faceted column-wise by the four five-year periods: 2000–2004 (red), 2005–2009 (green), 2010–2014 (blue), and 2015–2019 (purple).
